# Enhancement of Naringenin Bioavailability by Complexation with Hydroxypropoyl-β-Cyclodextrin

**DOI:** 10.1371/journal.pone.0018033

**Published:** 2011-04-06

**Authors:** Maria Shulman, Merav Cohen, Alejandro Soto-Gutierrez, Hiroshi Yagi, Hongyun Wang, Jonathan Goldwasser, Carolyn W. Lee-Parsons, Ofra Benny-Ratsaby, Martin L. Yarmush, Yaakov Nahmias

**Affiliations:** 1 Center for Bioengineering, School of Computer Science & Engineering, Institute of Life Sciences, The Hebrew University of Jerusalem, Jerusalem, Israel; 2 Center for Engineering in Medicine, Massachusetts General Hospital, Harvard Medical School, Boston, Massachusetts, United States of America; 3 Harvard-MIT Division of Health Science and Technology, Cambridge, Massachusetts, United States of America; 4 Children's Hospital Boston, Harvard Medical School, Boston, Massachusetts, United States of America; 5 Department of Chemical Engineering, Northeastern University, Boston, Massachusetts, United States of America; 6 Department of Biomedical Engineering, Rutgers University, Piscataway, New Jersey, United States of America; Biological Research Center of the Hungarian Academy of Sciences, Hungary

## Abstract

The abundant flavonoid aglycone, naringenin, which is responsible for the bitter taste in grapefruits, has been shown to possess hypolipidemic and anti-inflammatory effects both *in vitro* and *in vivo*. Recently, our group demonstrated that naringenin inhibits hepatitis C virus (HCV) production, while others demonstrated its potential in the treatment of hyperlipidemia and diabetes. However, naringenin suffers from low oral bioavailability critically limiting its clinical potential. In this study, we demonstrate that the solubility of naringenin is enhanced by complexation with β-cyclodextrin, an FDA approved excipient. Hydroxypropoyl-β-cyclodextrin (HPβCD), specifically, increased the solubility of naringenin by over 400-fold, and its transport across a Caco-2 model of the gut epithelium by 11-fold. Complexation of naringenin with HPβCD increased its plasma concentrations when fed to rats, with AUC values increasing by 7.4-fold and C_max_ increasing 14.6-fold. Moreover, when the complex was administered just prior to a meal it decreased VLDL levels by 42% and increased the rate of glucose clearance by 64% compared to naringenin alone. These effects correlated with increased expression of the PPAR co-activator, PGC1α in both liver and skeletal muscle. Histology and blood chemistry analysis indicated this route of administration was not associated with damage to the intestine, kidney, or liver. These results suggest that the complexation of naringenin with HPβCD is a viable option for the oral delivery of naringenin as a therapeutic entity with applications in the treatment of dyslipidemia, diabetes, and HCV infection.

## Introduction

In recent years, polyphenols, and flavonoids in particular, have emerged as a class of natural products shown to have anti-oxidant, anti-atherogenic, and normolipidemic effects [1]. One of the most abundant is the citrus flavonoid-glycoside naringin, which is responsible for the bitter taste in grapefruit. Naringin is hydrolyzed to naringenin by gut flora prior to being absorbed [2]. Naringenin has been widely studied, and has been reported to be an antioxidant [3], [4], MTP and ACAT inhibitor [5], [6], and a regulator of cytochrome P450 (CYP450) enzymes including, CYP1A, CYP3A4, and CYP4A [7], [8], [9]. The ability of naringenin, and its glucuronide metabolites, to reduce plasma cholesterol levels has been demonstrated *in vivo* [10], [11], [12], while its ability to reduce ApoB secretion has been demonstrated extensively *in vitro* [13], [14]. A recent clinical trial in hypercholesterolemic patients demonstrated that a 400 mg/day dose of naringin lowered LDL levels by 17% [3]. Similar cholesterol lowering effects of naringenin were demonstrated in rabbits [11] and rats [12]. More recently, Huff and coworkers have shown that naringenin helps correct many of the lipid disturbances associated with diabetes in transgenic mice lacking the LDL receptor that were fed a western-style diet, including correction of VLDL overproduction, amelioration of hepatic steatosis, and attenuation of dyslipidemia [10], while our group demonstrated that naringenin blocked the assembly of VLDL and infectious hepatitis C virus (HCV) particles in Huh7.5.1 cells and primary human hepatocytes [15].

Importantly, our recent findings demonstrate that naringenin is a dual-PPAR agonist, activating both PPARα and PPARγ through the induction of their co-activator PGC1α [16]. At the same time, naringenin directly inhibits LXRα, which controls HMG-CoA reductase (HMGR) expression in the liver [16]. These results suggest that naringenin could potentially replace the actions of fibrates (PPARα agonists), thiazolidenediones (PPARγ agonists), and statins (HMGR inhibitors) in the treatment of type-2 diabetes or hyperlipidemia [16].

Regretfully, the clinical relevance of naringenin is limited by its low solubility and minimal bioavailability owing to its largely hydrophobic ring structure. In this study, β-cyclodextrins were examined as potential excipients to enhance the solubility and enteral uptake of the flavonoid. Cyclodextrins are a family of cyclic oligosaccharides that create a 3-dimensional toroid structure, providing a cavity that can accommodate small hydrophobic molecules, such as cholesterol or steroids. Cyclodextrins can therefore be used as excipients to improve the solubility of hydrophobic drugs with similar structure [17], [18]. Specifically, the bioavailability of rutin, a flavonoid-glycoside similar in structure to naringin, was significantly enhanced by complexation with 2-hydroxypropyl-β-cyclodextrin (HPβCD) [19]. Here, we demonstrate that HPβCD enhances the solubility of naringenin, increases its transport across a Caco-2 model of human gut epithelium, and elevates its plasma concentrations following oral administration to Sprague-Dawley rats. When the complex is given right before a meal rich in glucose and fat, it decreased VLDL levels by 42% and increased the rate of glucose clearance by 64% compared to naringenin alone. These effects correlated with increased mRNA expression of the PPAR co-activator, PGC1α in both liver and skeletal muscle, strengthening recent evidence of a PPAR-mediated mechanism of action [16]. Combined with HPβCD's strong safety record, our results suggest that HPβCD-naringenin complexes could be used to efficiently deliver the flavonoid in patients for the treatment of dyslipidemia, arthrosclerosis, and HCV infection.

## Results

### β-Cyclodextrins increase the solubility of naringenin

**Figure 1:**
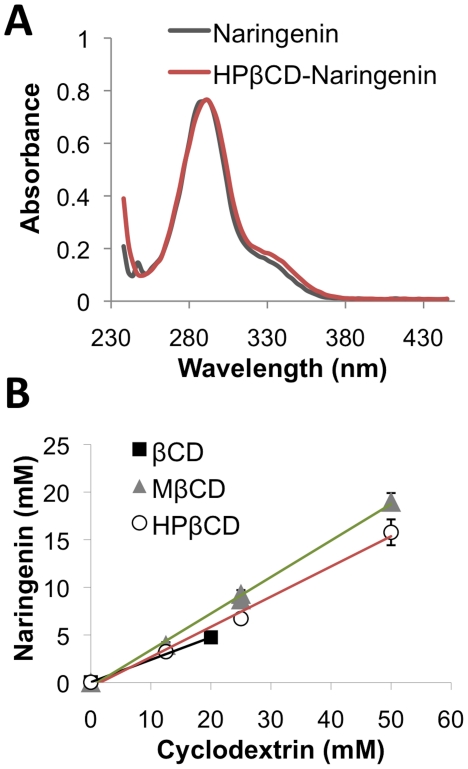
Cyclodextrins enhance the solubility of naringenin. (a) Complexation of naringenin with cyclodextrins resulted in minimal shift in the compound UV absorption spectrum. (b) Naringenin solubility in water was measured to be 36±1 µM, whereas its soluble fraction increased with the addition of βCD, mβCD, or HPβCD by 132-fold, 526-fold, and 437-fold, respectively.

Molecules similar to naringenin in structure and size were previously shown to be solubilized by complexation with β-cyclodextrin. To explore if naringenin is similarly solublized we generated complexes with β-cyclodextrin (βCD), methyl β-cyclodextrin (mβCD), and 2-hydroxypropyl-β-cyclodextrin (HPβCD). UV analysis indicated that complexation with cyclodextrins resulted in a very small shift in naringenin's absorption spectrum (**Figure 1A**). Concentrations of naringenin were then extrapolated from the previously obtained standard curve (**Figure 1C**). As expected, naringenin solubility in water was 36±1 µM, consistent with previously observed results [20]. Upon complexation with cyclodextrins, the amount of solubilized naringenin increased, as summarized in **Table 1**. The three βCDs solubilized naringenin in decreasing order mβCD > HPβCD > βCD resulting in a significant 526, 437, and 132-fold, enhancement in solubility respectively (p<0.01).

**Table 1:** Naringenin solubility in cyclodextrin solutions.

	Naringenin (mM)	Cyclodextrin (mM)	Fold increase in solubility	K
**βCD**	4.8±0.3	20±1.0	132	6025
**mβCD**	19.0±0.9	50±2.5	526	9975
**HPβCD**	15.8±1.4	50±2.5	437	8203

### HPβCD enhances the transport of naringenin across a Caco-2 monolayer

While mβCD was the most effective in enhancing the solubility of naringenin, its use is associated with soft tissue and kidney damage due to its detergent-like effect on membranes [21]. On the other hand, HPβCD does not cause hemolysis or irritation due to its low surface tension and is generally regarded as a safe excipient [22]. We therefore examined the ability of HPβCD to enhance the transport of naringenin across a monolayer of Caco-2 cells, an established model for drug transport across the human gut epithelium.

**Figure 2:**
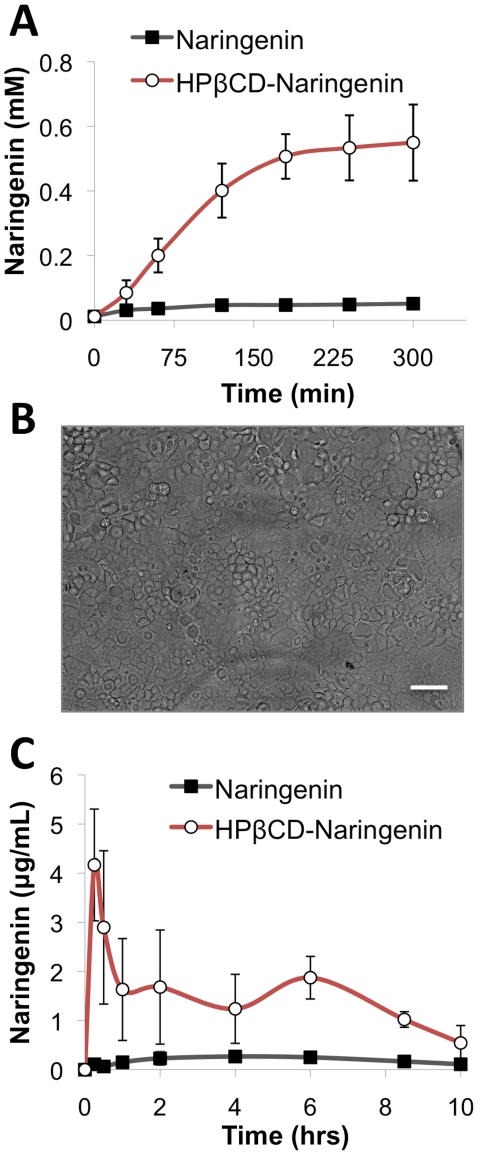
HPβCD enhances the bioavailability of naringenin. (a) 11 mM naringenin, either alone or in a complex with 45 mM HPβCD, was added to the top apical chamber of a Caco-2 model of human intestinal transport. In the presence of HPβCD, the concentration of naringenin was increased in the bottom basal side from 40±20 nM to 510±70 nM, representing an 11-fold enhancement of transport across the Caco-2 monolayer. (b) Phase image of Caco-2 monolayer grown for 21 days on collagen-coated transwell membranes. Barrier resistance remained unchanged during the course of the experiment. (c) Male Sprague-Dawley rats were fed 20 mg/kg body weight naringenin either alone, or as a HPβCD-naringenin complex. Blood samples were collected sequentially and analyzed for naringenin content by LC-MS. HPβCD-naringenin complex had higher oral bioavailability compared to naringenin alone. AUC_0-10_ of the HPβCD-naringenin complex increased by 7.4-fold (p = 0.005), and maximal concentration, C_max_ increased by 14.6-fold (p = 0.002).

Caco-2 cells were grown for 21 days on collagen-coated 1 cm^2^ porous transwell membranes (0.4 µm pores) on which cells formed differentiated monolayers, expressing major tight junction proteins, microvilli, and drug transporters (**Figure 2B**) [23]. Transepithelial Electrical Resistance (TEER) and Lucifer yellow transport were used to evaluate epithelial integrity and maturity of the monolayers. The apparent permeability coefficient, P_app_, remained between 6 and 7×10^−7^ cm/sec through the course of the experiment, demonstrating that the Caco-2 layer was intact. 11 mM naringenin, either alone or in a complex form with 45 mM HPβCD, was added to the top assay chamber. Samples were taken from both the top, apical chamber and the bottom, basal chamber at different time intervals and assayed for concentrations of naringenin (**Figure 2A**). In the presence of HPβCD, the concentration of naringenin at the basal chamber was increased from 0.04± 0.02 µM to 0.51± 0.07 µM, representing an 11-fold enhancement of transport across the Caco-2 monolayer. The integrity of the monolayer prior to and following the experiment was similar to control for both treatments.

### HPβCD enhances the bioavailability of naringenin in rats

To test whether cyclodextrin would enhance the oral bioavailability of naringenin, adult Sprague-Dawley rats were fed 20 mg/kg body weight naringenin either alone, or as a 1:16 (wt/wt) HPβCD-naringenin complex, using an oral gavage. Blood samples were collected sequentially for 10 hrs from the carotid artery using the previously placed catheter into tubes containing heparin. Immediately after collection, plasma was separated and stored at −80°C for further analysis. At the conclusion of the experiment, all animals were sacrificed, and liver, kidney, and bowel specimens were collected for histology. In an additional experiment, animals were placed in metabolic cages and urine was collected and pooled. Total naringenin (flavonoid and glucuronide) was determined by LC-MS as described above.

The complexation of HPβCD with naringenin significantly affected the plasma concentration versus time profile of the flavonoid (**Figure 2C**). Complexation with HPβCD significantly increased the AUC_0-10_ of naringenin from 2.0±0.5 hr×µg/ml to 15.0±4.9 hr×µg/ml representing a 7.4-fold increase in bioavailability (p = 0.005, n = 3). Naringenin's maximal concentration, C_max_, increased from 0.3±0.1 µg/ml to 4.3±1.2 µg/ml representing a 14.6-fold increase (p = 0.002, n = 3). The calculated half-life for naringenin in plasma remained unchanged in both conditions at 2.3 hours, consistent with values previously reported in humans [24], [25] and rats [26]. The percentage of free naringenin in plasma was in both cases <3% with the reminder in the glucuronide form. Finally, analysis of urine samples in two animals demonstrated unchanged renal clearance of 4.2 ± 1%.

### HPβCD–Naringenin complex reduces VLDL production and enhances glucose clearance following a lipid and glucose rich meal in rats

**Figure 3:**
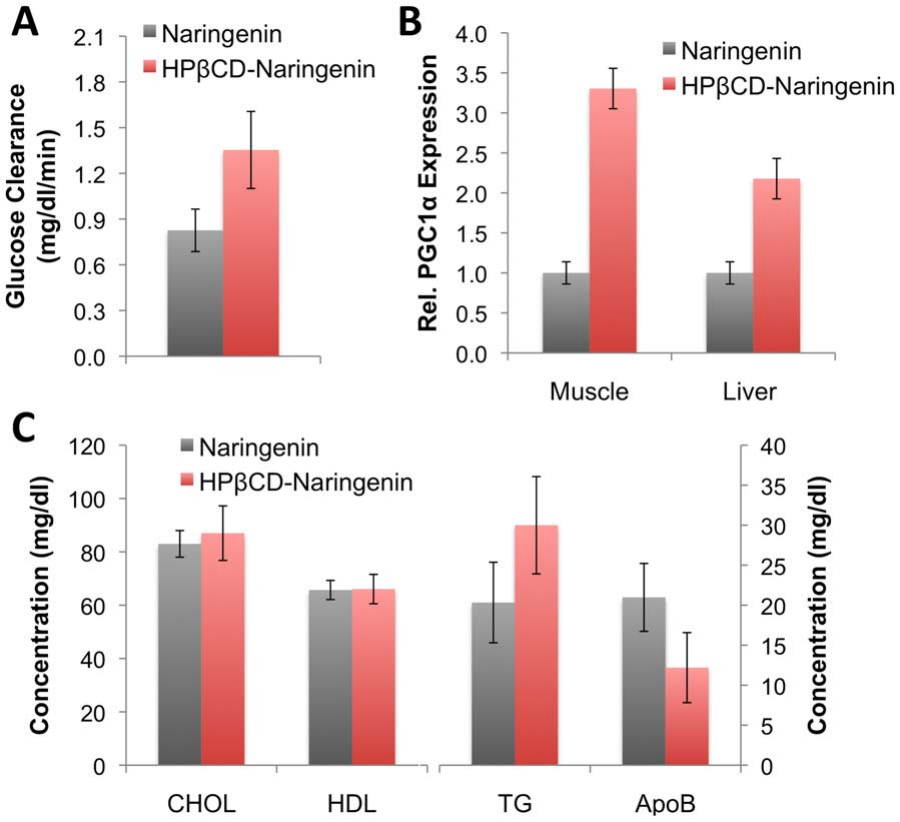
HPβCD-naringenin complex ameliorates the effects of a fat and glucose-rich meal. (a) Male Sprague-Dawley rats were fasted overnight, and then fed 20 mg/kg body weight naringenin either alone or as a HPβCD-naringenin complex. 30 min later, the rats were administered a meal composed of a suspension of 1 ml/kg olive oil and 1 g/kg glucose. Glucose clearance was measured as the rate of return to normal from maximal concentration (about 65 min). (b) Plasma cholesterol, HDL, triglycerides, and ApoB100 (VLDL) were measured 3.5 hrs after the meal. Cholesterol and HDL levels did not change. Triglyceride levels in rats fed the HPβCD-naringenin complex were elevated, but not significantly (p = 0.24, n = 3). In contrast, plasma levels of ApoB100, the structural protein of VLDL, were significantly 42% lower (p = 0.05, n = 3) than rats given naringenin alone. (c) mRNA abundance of PGC1α increased by 230±100% and 118±60% in skeletal muscle and liver, respectively. Tissue samples were collected 3.5 hrs after the meal.

To assess if a single dose of HPβCD–naringenin could affect rat metabolism we administered naringenin or its complex orally, 30 minutes before the oral administration of a meal high in lipids (1 ml/kg) and glucose (1 g/kg). Glucose levels were measured sequentially for 2 hrs after the meal (**Figure S1**). Interestingly, rats that were administered the HPβCD–naringenin complex showed a significantly 64% higher (p = 0.05, n = 3) rates of glucose clearance, compared to rats given naringenin alone (**Figure 3A**). Previous work showed that the maximal level of VLDL in blood is reached 3 to 4 hrs after a meal. Here we show that 3.5 hrs after the meal, plasma levels of ApoB100, the structural protein of VLDL were significantly 42% lower (p = 0.05, n = 3) then rats given naringenin alone (**Figure 3C**). Interestingly, triglyceride levels in the same rats increased, but not significantly (p = 0.24, n = 3). This response is similar to that of fibrates that, like naringenin, act through PPARα, and are thought to occur due to a flux of chylomicrons from the intestine.

Recently, we demonstrated that naringenin is a dual-PPAR agonist, activating both PPARα and PPARγ through the induction of their co-activator PGC1α [16]. To examine if naringenin acts through a similar mechanism *in vivo* we carried out qRT-PCR analysis on samples of liver and skeletal muscle taken 3.5 hrs after the meal. The expression of PGC1α significantly increased by 230±100% (p = 0.02, n = 3) and 118±60% in skeletal muscle and liver, respectively (**Figure 3B**).

### Oral administration of HPβCD-naringenin was not associated with adverse effects

**Figure 4:**
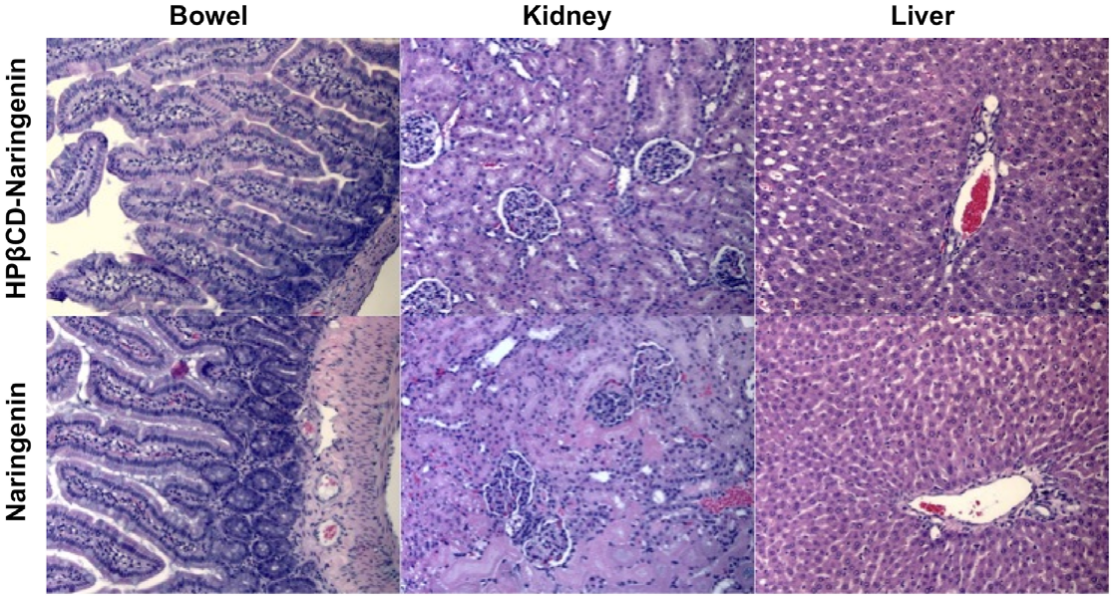
Male Sprague-Dawley rats were fed 20 mg/kg body weight naringenin either alone, or as a HPβCD-naringenin complex. Animals were sacrificed 10 hrs post-treatment and tissue samples were collected and preserved. Representative images of H&E histological preparations from bowel, kidney, and liver are presented. Tissues were evaluated by a blinded pathologist and were judged to be normal with no signs of inflammation or necrosis.

Lastly, we wished to examine if the administration of the HPβCD-naringenin complex was associated with tissue or organ damage. Liver, kidneys and intestine were removed 10 hrs following oral administration of the complex and showed no gross pathological changes (data not shown). Histological characterization by a blind observer demonstrated that the small intestine, kidney, and liver sections showed no evidence of tissue injury or inflammation in both groups. Liver sections showed no evidence of hepatocyte damage or neutrophil infiltration to the portal area, while kidney and intestine sections show no tubular/glomerular damage, edema or epithelial damage, respectively (**Figure 4**). One intestine section in a single rat showed a localized small infiltrate, which did not appear to be related to the experiment.

Comprehensive metabolic analysis was carried out on serum samples taken from rats, 10 hours after the treatment with HPβCD-naringenin, naringenin alone, as well as rats treated with saline as a control. The biochemical examination revealed no major changes (**Table 2**). Glucose and electrolytes levels were within normal values, as were urea and creatinine levels, suggesting kidney function was unchanged. Biochemical liver damage parameters were also within normal, with alkaline phosphatase (ALP) levels being actually lower in treated groups compared to control (p = 0.03), while ALT and AST showing no significant differences (p = 0.44 and p = 0.17, respectively). Total bilirubin (TBIL), albumin (ALB) and total protein (TP) content of the blood was also unchanged. Together with the histological and pathological analysis these results suggest that oral administration of HPβCD-naringenin complex was not associated with any adverse effects.

**Table 2:** Comprehensive Metabolic Panel of naringenin-treated male Sprague-Dawley rats compared with untreated controls.

	HPβCD-Naringenin	Naringenin	Control
**Na^+^**	139.0±1.0	138.0±1.0	134.3±2.5
**K^+^**	5.9±0.6	5.3±0.3	4.4±0.7
**Cl^-^**	102.0±1.0	102.0±2.0	89.7±3.9
**Glu**	168.0±14.4	157.0±6.1	181.2±26.5
**Ca^2+^**	9.3±0.7	9.7±0.2	10.2±0.5
**BUN**	16.7±3.1	19.3±5.1	14.0±3.6
**Cre**	0.2±0.0	0.3±0.0	0.4±0.1
**ALP**	147.3±84.6	135.3±37.5	311.7±64.6
**ALT**	56.7±11.1	61.3±20.3	59.8±30.4
**AST**	307.3±107.8	248.3±45.5	205.7±124.0
**TBIL**	0.3±0.0	0.3±0.1	0.4±0.0
**ALB**	1.6±0.2	1.6±0.3	2.1±0.1
**TP**	5.6±0.5	5.6±0.5	6.2±0.3

Glu, Glucose; BUN, blood urea nitrogen; Cre, Creatinine; ALP, alkaline phosphatase; ALT, alanine transaminase; AST, aspartate transaminase; TBIL, total bilirubin; ALB, albumin; TP, total protein.

## Discussion

Naringin is an abundant flavonone-glycoside known to cause the bitter taste in grapefruit (*citrus paradisi*). The compound is highly soluble and can be found in grapefruit juice at concentrations of up to 0.5 g/L [26]. Following ingestion, naringin is broken down by gut flora to its aglycone form, naringenin [27]. Naringenin has been the focus of multiple studies in recent years, which began to elucidate its clinical potential as an antioxidant with anti-carcinogenic, anti-inflammatory, and hypolipidemic properties [2]. The flavonoid's ability to reduce the secretion of very-low density lipoprotein (VLDL) from hepatocytes has been demonstrated in tissue culture, and attributed both to signaling events, through the insulin-PI3K and MAPK pathways [6], [28]; and most recently, to the modulation of the PPARα, PPARγ and LXRα nuclear receptors [16], [29]. Recently, our group demonstrated that this inhibition of VLDL assembly blocked the production of infectious HCV particles from infected hepatocytes [15], while others point to naringenin's hypolipidemic properties in the treatment of diabetes [10]. As with other drugs, efficacy would depend on the ability to deliver the molecule to patients in a reproducible manner [21].

Cyclodextrins are a family of cyclical oligosaccharides, composed of varying numbers of glucopyranoside rings that form a three-dimensional toroid structure. The inner face of the toroid is significantly less hydrophilic than the surrounding water, providing an energetic advantage to the insertion of hydrophobic molecules into the cavity. β-cyclodextrins, specifically, are composed of seven sugar rings, and have been shown to be non-toxic to humans [21]. These cyclodextrins are widely used by the food and pharmaceutical industries and a generally regarded as safe [22]. The potential of β-cyclodextrins to enhance the solubility and gut absorption of flavonoids was demonstrated by Uekama and coworkers [19]. The group complexed the flavonoid-glycoside, rutin with HPβCD and found a 10-fold increase in solubility. Following the oral administration of the complex in beagle dogs the plasma concentration of rutin increased by nearly 3-fold. The hydrophobic nature of naringenin, and its structural similarity to the quercetin unit in rutin, suggests that its delivery could similarly be enhanced.

Naringenin suffers from low solubility in aqueous environments, up to 36 µM in our hands, and is generally dissolved in organic solvents [2]. In the presence of β-cyclodextrins, however, the solubility of naringenin increased by several orders of magnitude, up to 500-fold. Of the three cyclodextrin types tested, solubility increased in the order mβCD>HPβCD>βCD. Despite the superior ability of mβCD to solubilize naringenin, we chose to conduct further experiments with HPβCD, which does not exert a detergent-like effect on biological membranes causing irritation and hemolysis [30] and is used in multiple drug formulations [31].

We next examined the ability of HPβCD to enhance the delivery of naringenin across the intestinal mucosa. We used the well-characterized Caco-2 transwell model of the human gut epithelium [23]. In this experiment, a monolayer of Caco-2 gut epithelial cells was grown on a transwell membrane, and the ability of naringenin to cross this barrier is measured over time. When complexed to HPβCD, naringenin reached a concentration 11-fold higher than in the absence of the excipient. Interestingly, the rate of transport of naringenin across the membrane was not different between the groups, set as 5±1 µM/min (p>0.10). The integrity of the monolayer was verified both at the beginning and end of the experiment suggesting that neither HPβCD nor naringenin damaged the monolayer at the concentrations and time-scales examined.

We next examined the ability of HPβCD to enhance the bioavailability of naringenin in a rat model. Two groups of male Sprague-Dawley rats were fed 20 mg/kg body weight naringenin. One group was fed naringenin alone, while the other was fed a HPβCD-naringenin complex. Our results indicate a substantial improvement in the delivery of naringenin complexed with HPβCD, with AUC_0-10_ of naringenin increasing 7.4-fold and maximal concentration, C_max,_ increasing 14.6-fold over naringenin alone. This increase in bioavailability represents an increase in the absorption rate from K_a_  = 63.7 hr^−1^ to K_a_  = 26.9×10^4^ hr^−1^, a 4200-fold increase. Several effects could explain this increased rate of transport, including enhancement of dissolution kinetics, increase in solubility, decrease in degradation, change in the properties of the intestinal membrane, and shuttling and enhancement of drug concentration at the intestinal wall [31]. However, it is unlikely that complexation with HPβCD changes the plasma pharmacokinetics of naringenin, as cyclodextrins are poorly transported across the intestinal wall [21]. The calculated half-life for naringenin in plasma under both conditions was 2.3 hrs, consistent with values previously reported in humans [24], [25] and rats [26]. The ratio of free naringenin to its glucuronide form were also unchanged by the complex and remained <3% in both cases.

Using this information we devised a study in which naringenin or the HPβCD-naringenin complex is given orally to rats 30 min prior to a controlled meal rich in glucose and fat. This 30 min period was judged sufficient to allow the flavonoid to induce PPARα in liver and skeletal muscle through our recently described induction of the PPAR co-activator PGC1α [16]. We show that animals which received the complex showed significantly 64% higher (p = 0.05, n = 3) rates of glucose clearance, compared to rats given naringenin alone. Correspondingly, skeletal muscle expression of PGC1α measured by qRT-PCR significantly increased by 230±100% (p = 0.02, n = 3). In addition, 3.5 hrs after the meal, plasma levels of ApoB100, the structural protein of VLDL were significantly 42% lower (p = 0.05, n = 3) in rats given the complex than rats given naringenin alone. Not surprisingly, the expression of PGC1α in the liver was also increased by 118±60%. Interestingly, triglyceride levels in the complex-fed rats increased, but not significantly (p = 0.24, n = 3). This response is similar to that of fibrates that, like naringenin, act through PPARα, and is thought to occur due to a flux of chylomicrons from the intestine being ‘ignored’ by the liver.

Previous studies demonstrated the low bioavailability of naringenin. Niopas and coworkers orally administered 135 mg naringenin to six healthy volunteers. Plasma concentrations peaked after 3.5 hrs, and bioavailability was estimated to be 5.8% [24]. Erlund and coworkers found similarly low bioavailability when the source of naringenin was grapefruit juice. The researchers also noted the high variability in bioavailability, which was hypothesized to be the result of subject-to-subject variation in gut microflora [25]. Importantly, plasma concentration of naringenin attained in these trials is significantly lower than the concentration required to attain a therapeutic effect, measured both *in vitro* and *in vivo*. Huff and colleagues have demonstrated that in HepG2 cells naringenin peak modulation of lipid metabolism is attained around 200 µM [5]. A similar concentration of naringenin blocked the production of VLDL and HCV in chronically infected Huh7.5.1 cells and primary human hepatocytes [15]. Our recent findings regarding the modulation of PPARα and LXRα suggest a *switch-like response* to naringenin at concentrations of around 150 µM [16] partly explaining why animal experiment resort to either very high doses or several weeks of treatment to demonstrate an effect.

Attaining plasma concentrations of 150–200 µM requires the consumption of more than 5 g of naringenin, over 60 grapefruits worth, at 5.8% bioavailability. In contrast, based on our work, less than 400 mg of naringenin are required if the compound is complexed with HPβCD. Considering the sugary taste of cyclodextrin, it is no longer such a bitter pill to sallow.

## Materials and Methods

### Ethics Statement

All animals were treated in accordance with National Research Council guidelines and approved by the Subcommittee on Research Animal Care at the Massachusetts General Hospital and the Hebrew University of Jerusalem. Experiments were approved under IACUC protocol numbers 2009N000171 and NS-10-12489-3 in the United States and Israel, respectively.

### Materials

Naringenin, β-cyclodextrin (βCD), methyl β-cyclodextrin (mβCD), and 2-hydroxypropyl-β-cyclodextrin (HPβCD) were purchased from Sigma-Aldrich Chemicals (St. Louis, MO). Caco-2 human epithelial colorectal adenocarcinoma cells were purchased from the American Type Culture Collection (Rockville, MD). Unless otherwise noted, all other chemicals were purchased from Invitrogen Life Technologies (Carlsbad, CA).

### Solubility curves of naringenin complexed with cyclodextrin

Stock solutions of naringenin were prepared in ethanol. A calibration curve was prepared by measuring the UV absorbance of the naringenin stock solutions (0.1 to 0.6 mM) at 290 nm using a ND-1000 spectrophotometer (NanoDrop Technologies, Rockland, DE). Standard deviations between triplicate measurements were less than 5%.

Improvements in naringenin solubility when complexed with cyclodextrin were determined and evaluated as follows; stock solutions of βCD, mβCD, and HPβCD were prepared in distilled water. None of the cyclodextrins absorbed at 290 nm for concentrations from 0 to 50 mM (data not shown). Next, excess amounts of naringenin powder were added to solutions containing variable amounts of each cyclodextrin, vortexed, and incubated with shaking at 37°C for 3-5 hrs. Naringenin-cyclodextrin solutions were filtered through a 0.45 µm filter to remove the undissolved naringenin, diluted by 20 or 50-fold, and absorbance was measured at 290 nm. The complex stability constant K was calculated from the linear portion of the solubility diagram assuming a 1:1 complex.

### Caco-2 cell culture

Caco-2 cells were cultured in Dulbecco's Modified Eagle's Medium (DMEM) supplemented with 10% fetal bovine serum, 1% nonessential amino acids, and 4 mM glutamine without antibiotics. The cultures were grown in a humidified incubator at 37°C and 5% CO_2_. Cells between 30 and 53 passages were used.

### Intestinal transport assay

For the transport studies, Caco-2 cells were seeded on Transwell (0.4-µm pore size, 1-cm^2^ growth area; Corning Costar Co.) at a cell density of 1x10^5^ cells/filter. Cell growth and maintenance were performed as previously described [32]. The cell monolayer was fed fresh growth medium every 2 days and used on day 21 for the transport experiments. HBSS supplement with 20 mM glucose and 10 mM HEPES (pH 7.35) was used as the transport medium. To determine the amount of drug crossing the polarized Caco-2 cell monolayer from the donor to the receiver (i.e., apical to basolateral), the Caco-2 cells were rinsed twice with pre-warmed transport medium and incubated by pre-warmed transport medium 0.2 ml for apical chamber and 0.5 ml for basolateral chamber at 37°C for 30 min. A 60 mg/ml (1% DMSO in HBSS) stock solution of test compounds, either naringenin or HPβCD-naringenin, was added and samples from both apical and basolateral were taken (30 µl) at different time points: 30, 60, 120, 150 180, 240, and 300 min. The integrity of the culture was confirmed by transepithelial electrical resistance (TEER) and by detecting fluorescently labeled cells using 60 µM of Lucifer Yellow as a standard. The concentrations of naringenin or HPβCD-naringenin were determined as described and plotted as a concentration on the basolateral side vs. time. Concentrations were corrected by the dilution factor as fresh buffer was added after sampling.

### Animal experiments

Adult male Sprague-Dawley rats were purchased from Charles Rivers Laboratories (Wilmington, MA). Upon arrival, each rat was isolated for 3–5 days towards adaptation to the new environment. Animals were housed under 12h cycle of day/night with free access to drinking water and fed *ad libitum* unless otherwise noted.

To measure the pharmacokinetic profile of naringenin, rats weighing between 280 and 300 g were anaesthetized using intraperitoneal injections of ketamine and xylazine at 110 and 0.4 mg/kg, respectively. The left carotid artery was cannulated using a 0.76-mm diameter ×60-cm length heparanized catherter. The catheter was tunneled subcutaneously from the opening made in the anterior face of the neck to the dorsal site of the neck and permanently anchored in the skin. The catheter was secured by the use of a rat jacket. Animals were placed in their cages during the term of the study. Animals were orally administered with 20 mg/kg body weight of naringenin in either water or complexed with 320 mg/kg body weight HPbCD using a rat oral gavage (18G ×1 1/2″ plastic feeding tube from Instech Laboratories, Inc, PA, USA). Blood samples (0.5 ml) were collected at 0, 15, 30, 60, 120, 240, 360, 510, and 600 min from the carotid artery using the previously placed catheter. In two additional experiments, animals were placed in metabolic cages and urine was collected and pooled for the duration of the experiment.

To measure metabolic changes, rats weighing between 270 to 290 g were fasted for 16 hrs, but allowed free access to water. Animals were orally administered with 20 mg/kg body weight of naringenin in either water or complexed with 320 mg/kg body weight HPbCD using a rat oral gavage. Precisely 30 min after the oral administration of naringenin, the rats were administered 1 ml/kg of olive oil suspended in PBS with 1 g/kg of glucose using rat oral gavage. Glucose was measured using a single tail snip and repeated scratching on an Accu-Chek Sensor (Roche, Branford, CT) prior to the experiment and at 0, 15, 30, 60, 90, and 120 min from the meal. Rats were anaesthetized 200 min following the meal using intraperitoneal injection of ketamine and xylazine followed by terminal blood draw and tissue collection.

### LC-MS detection of naringenin

LC-MS analysis was performed on an Agilent Technologies series 1100 LC-MSD system (Santa Clara, CA), which included an Agilent 1100 quaternary pump, autosampler, column oven, on-line vacuum degassor, and single quadrupole mass spectrometer equipped with electrospray ion source (ESI).

Mass spectrometry conditions: Electrospray ionization (ESI), positive, selected ion monitoring scan (SIM); SIM: naringenin m/z 273.1. LC conditions: Eclipse XDB-C18 column (4.6×150mm, 5.0 µm). The mobile phase was composed of methanol-water with 0.1% formic acid (65:35,v/v). The isocratic flow rate was set at 0.8 ml/min and injection volume was only 10 µl.

To each 100 µl of rat serum sample, 100 µl of 0.1N sodium acetate (pH = 5.0) and 100 µl of β-glucuronidase enzyme (5000 units/ml, type HP-2 from Helix Pomatia) were added and vortexed for 5 seconds. This process hydrolyzes the conjugated form of naringenin to determine total naringenin in plasma. After addition of 20 µl IS buffer solution (5 µg/ml), the sample was then incubated at 37°C water bath for 18 h.

The sample was extracted with 0.8 ml of ethyl acetate after 18 h incubation, and centrifuged at 13000 rpm for 10 min. The supernatant was collected and evaporated to dryness under nitrogen at room temperature. The residue was reconstituted with 100 µl of mobile phase and filtered through a micro nylon n filter (0.45 µm). 10 µl of the filtrate was forwarded to LC-MS analysis. A calibration curve was established and QC samples conducted (data not shown). Data acquisition was performed using ChemStation software (Agilent). Linear regression (weighted by 1/x) between serum concentration and peak area ratio of naringenin to IS was constructed using SPSS11.0 statistical software. The concentrations of naringenin in samples were calculated by interpolation of the linear equation.

### Quantitative Reverse Transcription Polymerase Chain Reaction (qRT-PCR)

Liver and skeletal muscle tissues were ground in liquid nitrogen, homogenized and RNA was purified using BioRad Aurum Total RNA Fatty and Fibrous Tissue Kit (Hercules, CA). Total RNA was quantified on NanoDrop Technologies, ND-1000 spectrophotometer (Wilmington, DE) and mRNA transcript abundance was measured on a BioRad CFX96 real-time PCR Detection System using Bio-Rad SsoFast™ EvaGreen® supermix (Hercules, CA), according to the manufacturers' instructions. PGC1α (PPARGC1a) primers used were, forward GACCCCAGAGTCACCAAATGA and reverse GGCCTGCAGTTCCAGAGAGT, while β-Actin (ACTB) used were, forward AGCCATGTACGTAGCCATCCA and reverse TCTCCGGAGTCCATCACAATG (Integrated DNA Technologies, Coralville, IA). Expression was normalized to β-Actin using ΔΔCt.

### Metabolism

Liver metabolic and lipid plasma levels were analyzed using Piccolo Blood Analyzer (Abaxis, Union City, CA) and confirmed by A.M.L veterinary department (Herziliya, Israel). Levels of rat apolipoprotein B100 (APOB100) were measured using ELISA (Uscn Life Sciences, Wuhan, China).

### Liver Histology

Histological sections of each organ were taken 10 hours after treatment. Formalin-fixed, paraffin-embedded liver, intestine, and kidney samples were sectioned at 4 µm and stained with hematoxylin & eosin (H&E). Histological characterization was performed by a blinded observer using standard assessment of damage.

### Statistics

Data are expressed as the mean ± standard deviation. Statistical significance was determined by a one-tailed Student's t-test. A *P*-value of 0.05 was used for statistical significance.

## Supporting Information

Figure S1 Glucose plasma concentrations over time in rats administered a high fat (1 ml/kg) high glucose (1 g/kg) meal at time zero. 30 minutes prior to the meal, the rats were administered either naringenin alone or HPβCD-naringenin complex. Curves represent average ± standard deviation of 3 rats in each group.
(PDF)
