# Correction: Enhancement of Naringenin Bioavailability by Complexation with Hydroxypropoyl-β-Cyclodextrin

**DOI:** 10.1371/annotation/c5a7bd78-0cd2-4575-a9fe-6729b2c2a217

**Published:** 2012-10-17

**Authors:** Maria Shulman, Merav Cohen, Alejandro Soto-Gutierrez, Hiroshi Yagi, Hongyun Wang, Jonathan Goldwasser, Carolyn W. Lee-Parsons, Ofra Benny-Ratsaby, Martin L. Yarmush, Yaakov Nahmias

The word "Hydroxypropyl" is misspelled in the article title. The correct title is: Enhancement of Naringenin Bioavailability by Complexation with Hydroxypropyl-β-Cyclodextrin.

The correct citation is: Shulman M, Cohen M, Soto-Gutierrez A, Yagi H, Wang H, et al. (2011) Enhancement of Naringenin Bioavailability by Complexation with Hydroxypropyl-β-Cyclodextrin. PLoS ONE 6(4): e18033. doi:10.1371/journal.pone.0018033

Hydroxypropyl is also misspelled in the fifth sentence of the abstract. The fifth sentence should read: Hydroxypropyl-β-cyclodextrin (HPβCD), specifically, increased the solubility of naringenin by over 400-fold, and its transport across a Caco-2 model of the gut epithelium by 11-fold.

