# Supplementary figures and images for: Enhancement of Naringenin Bioavailability by Complexation with Hydroxypropoyl-β-Cyclodextrin
Source: PLoS One. 2011 Apr 6;6(4):e18033. doi: 10.1371/journal.pone.0018033 (PMC3071816; doi:10.1371/journal.pone.0018033)

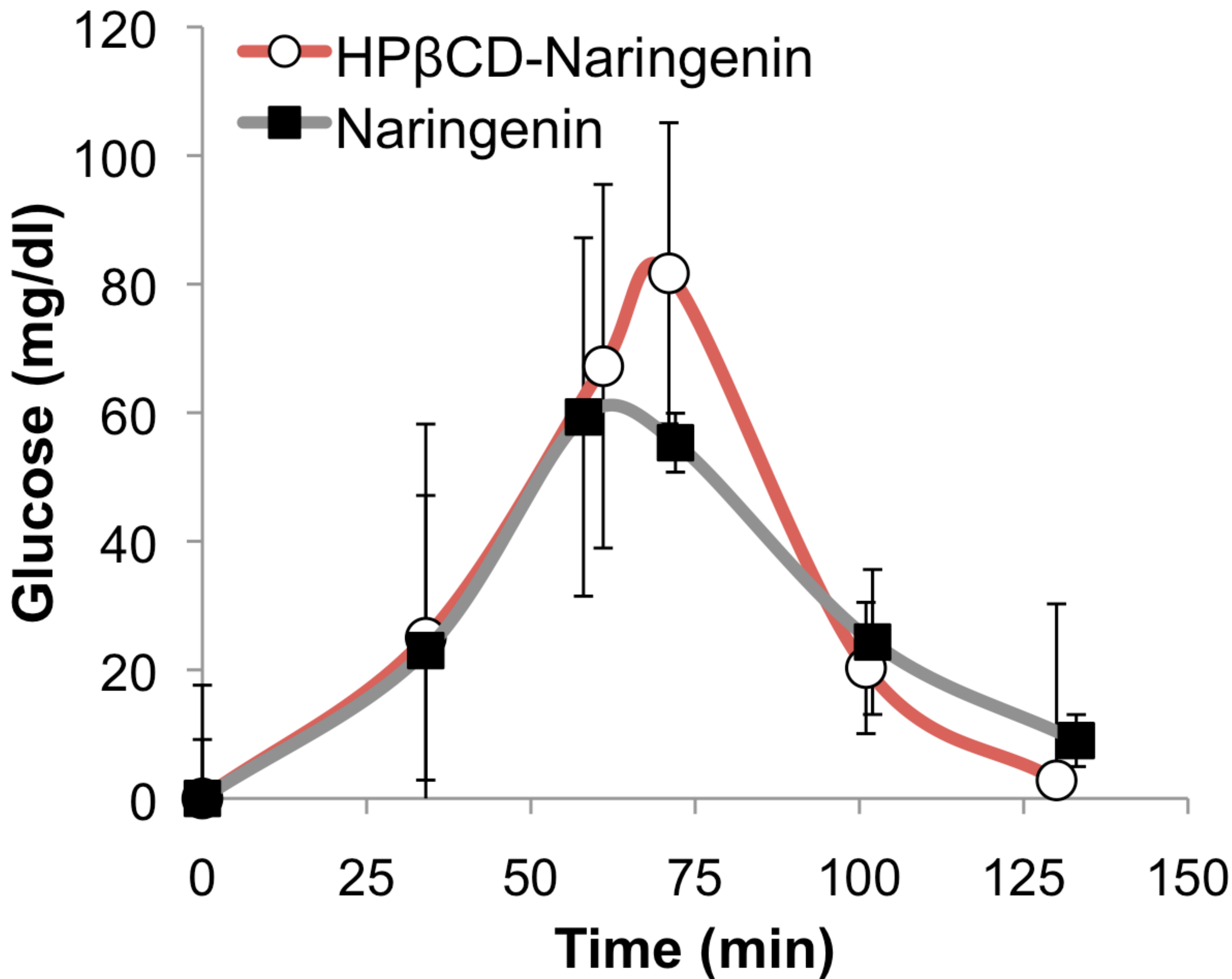

Supplement: Figure S1 — Glucose plasma concentrations over time in rats administered a high fat (1 ml/kg) high glucose (1 g/kg) meal at time zero. 30 minutes prior to the meal, the rats were administered either naringenin alone or HPβCD-naringenin complex. Curves represent average ± standard deviation of 3 rats in each group. (PDF) [file pone.0018033.s001.pdf]
